# The Combined Effect of the Association of Extractives from *Schinus lentiscifolius* on the Fungus *Beauveria bassiana* Against the Tick *Rhipicephalus microplus*

**DOI:** 10.3390/pathogens15101058

**Published:** 2026-10-07

**Authors:** Rafaela Regina Fantatto, Yousmel Alemán Gainza, Luis Adriano Anholeto, Francesco Castrogiovanni, Rodrigo Sorrechia, Alessandro Pelegrine Minho, Ana Carolina de Souza Chagas, Rosemeire Cristina Linhari Rodrigues Pietro

**Affiliations:** 1 Department of Drugs and Medicines, São Paulo State University UNESP, Rodovia Araraquara-Jaú Km 1, Araraquara 14800-903, São Paulo, Brazil; 2 Department of General and Applied Biology, Institute of Biosciences, São Paulo State University, Rio Claro 13506-900, Sao Paulo, Brazil; yousmel@gmail.com; 3 Chemistry Department, Acadia University, Wolfville, NS B4P 2R6, Canada; lanholeto@outlook.com; 4 Department of Agriculture, Food and Environment, University of Catania, Premises in via Santa Sofia, 100-95123 Catania, Italy; fcastrogiovanni.agro@gmail.com; 5 EMBRAPA—Brazilian Agricultural Research Corporation, Southeast Livestock Unit, Rodovia Washington Luiz, km 234, São Carlos 13560-970, São Paulo, Brazil; alessandro.minho@embrapa.br (A.P.M.);

**Keywords:** *R. microplus*, parasites, entomopathogenic, *Schinus lentiscifolius*

## Abstract

The cattle tick *Rhipicephalus microplus* causes substantial economic losses worldwide and has developed resistance to several synthetic acaricides. Therefore, the search for sustainable alternatives for tick control has intensified. Plant-derived compounds and entomopathogenic fungi, such as *Beauveria bassiana*, represent promising candidates for integrated control strategies. This study evaluated the in vitro effects of *Schinus lentiscifolius* extracts of different polarities, used alone or in combination with *B. bassiana*, against different developmental stages of *R. microplus*. The activity of the plant extracts, fungal conidia, and their combinations was assessed using adult immersion, larval packet, and larval immersion assays. The combined treatments increased larval mortality compared with the extracts used alone in both larval assays. In the larval packet and larval immersion assays, the crude ethanolic extract of *S. lentiscifolius* combined with *B. bassiana* achieved 100% mortality at 20 mg/mL. However, the effects observed against engorged females were limited. These findings indicate that the combination of *S. lentiscifolius* extracts and *B. bassiana* has promising in vitro larvicidal activity against *R. microplus*. Further studies are needed to characterize the interaction between the agents and to evaluate their efficacy and safety under in vivo and field conditions.

## 1. Introduction

Damage resulting from infestation by the *R. microplus* tick is characterized by blood loss, anemia, appetite suppression, decreased immune response, reduced milk and meat production, damage to the leather, and the transmission of hemoparasites that cause bovine babesiosis, anaplasmosis, and other diseases that cause high mortality [1,2]. Ticks cause significant economic losses worldwide and in Brazil [3,4]. The market for acaricidal substances in Brazil represents a substantial source of financial activity. A study conducted by the National Union of the Animal Health Products Industry [5] indicates that the revenue of the animal health industry in Brazil is expected to grow by approximately 7.9% in 2025, reaching R$ 12.8 billion. Despite this large volume of trade and investment in the veterinary products market, there are still relatively few chemical molecules available without reports of resistance, highlighting a persistent gap in effective control methods. Therefore, several studies have been conducted in search of alternative approaches that are less harmful to animals and the environment, with particular emphasis on the use of plants and microorganisms. A little-studied and very promising family is Anacardiaceae; species of this family have already demonstrated known insecticidal activities, such as *Schinus terebinthifolius*, which showed potential against *Hypothenemus hampei* (coffee borer) [6]. Essential oils and leaf extracts from *Schinus molle* and *Schinus terebinthifolius* demonstrate promising acaricidal activity against multiple tick and mite species, with mortality rates reaching 90–100% [7], and the species *Schinus molle* has already shown insecticidal action against the tick *R. microplus* [8]. The species *Schinus lentiscifolius* (March.) D. C., popularly known as Aroeira cinzenta, is a species native to some countries in South America such as Uruguay, Paraguay, Argentina, Peru, and Brazil, being very common and used in popular medicine in Rio Grande do Sul [9]. This species has already shown tick-killing activity in in vitro screening carried out by Minho et al. 2020. Among 35 plant extracts evaluated in the Pampa Biome, the hexane extract was effective on *R. microplus* larvae and adults at a concentration of 50 mg/mL and had a repellent effect [10]. In recent decades, these entomopathogens have also been studied for tick control [11]. Strains of *B. bassiana* have demonstrated potential against different ectoparasites of veterinary importance, such as the tick *Amblyomma cajennense* [12], *Rhipicephalus sanguineus* [13], and *R. microplus* [14]. Therefore, the extracts were combined with B. bassiana conidia to investigate whether the combined treatment could enhance acaricidal activity. Although previous studies have demonstrated the acaricidal potential of *S. lentiscifolius* extracts and the pathogenicity of *Beauveria bassiana* against ticks, little is known about the effect of their combined use against *R. microplu*s. Therefore, this study aimed to evaluate the acaricidal potential of *S. lentiscifolius* extracts associated with *B. bassiana* conidia against *R. microplus*, investigating whether this combination could enhance efficacy and reduce the effective concentration required for tick control.

## 2. Materials and Methods

### 2.1. Plant Material

The species *S. lentiscifolius* was provided by EMBRAPA CPPSUL under the responsibility of Dr. Alessandro P. Minho and collected at coordinates 31°33′ S lat, 54°01′ W. The species was registered in SISGEN under number AD73F75.

### 2.2. Production and Fractionation of Plant Extracts

The plant material was dried in an oven with air circulation at 40 °C and subsequently ground in a knife mill.

The ethanolic extract was obtained from the aerial parts of *S. lentiscifolius* using the 1:10 maceration method in absolute ethanol. After 7 days, the content was filtered, rotary evaporated, and placed in an amber bottle. Fractionation was carried out using the liquid–liquid partition method with high-, medium-, and low-polarity solvents (ethyl acetate, hexane, and water). Then, 2 g of ethanolic extract (SLb) was solubilized in 200 mL of ethyl acetate, centrifuged, and the supernatant was placed in a separation funnel together with 100 mL of distilled water. It remained like this for 45 min and was stirred every 15 min, removing and adding water three times (SLac).

Afterwards, this acetate fraction was rotary evaporated, resuspended, and placed in a separation funnel together with 50 mL of hexane (SLh). Afterwards, both fractions were rotary evaporated and placed in a beaker at a temperature of –80 °C. The last fraction was obtained from water resuspension (SLw).

### 2.3. Fungus Cultivation Conditions

*B. bassiana* strain 487 used in the tests was obtained from the Embrapa Cenargen Collection and registered in the ARSEF (Agricultural Research Service Collection of Entomopathogenic Fungal Cultures) database. Cultures were maintained, and conidia were produced on commercial potato dextrose agar (PDA) and incubated at 28 °C for 7 days.

### 2.4. Preparation of the Fungal Suspension for Testing

To prepare the fungal suspension, the conidia were scraped from the culture plates and transferred to 0.09% saline solution. The conidia concentration was determined using a Neubauer chamber, and the suspension was adjusted to a final concentration of 10^6^ conidia/mL. Then, RPMI 10% was added to the final suspension as a nutrient source for fungal activity, maintaining the final concentration at 10^6^ conidia/mL.

### 2.5. Positive Control

Using the commercial product (410 mg/mL), a 1:801 (*v*/*v*) dilution (as specified in the Colosso Pulverização^®^ (Ourofino Saúde Animal Ltda., Cravinhos, SP, Brazil) package insert) was prepared in distilled water, yielding a concentration of 0.512 mg/mL.

### 2.6. Immersion Test of R. microplus Females

Engorged females of *R. microplus* were collected from naturally infested cattle at Embrapa Pecuária Sudeste, São Carlos, SP, Brazil (21°57′42″ S, 47°50′28″ W). According to a resistance test performed in 2016, the ticks from this herd were resistant to pyrethroids, organophosphates, and amidines.

The tests were conducted following the immersion test of the engorged female, as described by [15]. Drummond et al. (1973) used three homogeneous groups of 10 engorged females per treatment/concentration.

The concentrations of the extract/fungus associations evaluated were determined using MFC, and it was found to be safe to start at 20 mg/mL, which corresponded to half of the Minimum Inhibitory Concentration determined for the fungus. The tests also included a negative control, solvent control (Tween 2%), and fungus control (10^6^ conidia/mL). The engorged females in the test groups were immersed in the substances to be tested for 5 min. They were then dried and placed in a Petri dish for oviposition in an incubator at an average temperature of 27 °C and relative humidity greater than 80% for 18 days. After the eighteenth day, oviposition was evaluated, with the eggs weighed and placed in adapted disposable syringes and kept in an incubator at 27 °C for another 18 days.

The parameters were calculated according to Drummond et al. (1973) [15] using the following equation:

Oviposition index (OI)

OI (%) = [egg mass (g)/mass of engorged females (g)] × 100

Percentage reduction in oviposition

Oviposition reduction (%) = [(OIcontrol − OItreated)/OIcontrol] × 100

Percentage reduction in hatchability

Hatchability reduction (%) = [(Hcontrol − Htreated)/Hcontrol] × 100

Reproductive efficiency (RE)

RE = [egg mass (g) × (hatchability (%)/100) × 20,000]/mass of engorged females (g)

Product effectiveness

Product effectiveness (%) = [(REcontrol − REtreated)/REcontrol] × 100

where OIcontrol and OItreated represent the oviposition indices of the control and treated groups, respectively; Hcontrol and Htreated represent the hatchability values of the control and treated groups, respectively; and REcontrol and REtreated represent the reproductive efficiency values of the control and treated groups, respectively. The results were determined and expressed as mean ± standard deviation.

### 2.7. Sensitivity of Larval Packet Test on Impregnated Paper

Sensitivity tests of *R. microplus* larvae were performed according to the technique recommended by FAO (1971) [16]. In this methodology, approximately 100 larvae from engorged females collected at Embrapa Pecuária Sudeste were placed on filter paper measuring approximately 10 × 8 cm, impregnated with different concentrations of samples or controls. Each of these papers impregnated with extractives and/or *B. bassiana* at a concentration of 10^6^ conidia/mL was folded, forming a “sandwich,” and sealed with plastic nails. The packets were maintained in a BOD incubator at 27 °C and 80% relative humidity. The reading took place after 7 days of incubation with the help of a vacuum compressor, adapted with a pipette, differentiating live and dead larvae.

### 2.8. Larvae Immersion Test

Around 300 larvae were added to 1 mL of each concentration of each solution to be tested, containing plant extractives and/or *B. bassiana* fungus 10^6^ conidia/mL. Triton^®^ (Hexis Científica, Indaiatuba, SP, Brazil) (0.02%) was added to this solution to ensure the immersion of the larvae. These larvae were immersed for 10 min. After the time elapsed, the larvae were placed in filter paper packets in three repetitions of 100 larvae [17]. These packets were sealed and stored in a BOD incubator at 27 °C and 80% relative humidity for 48 h, and after this period the test was read, differentiating live larvae from dead larvae with the aid of a vacuum compressor.

### 2.9. Statistical Analyses

Data were expressed as mean ± standard deviation. Differences among treatments were evaluated by one-way analysis of variance (ANOVA), followed by Tukey’s significant difference test. Statistical significance was set at *p* < 0.05. Analyses were performed using ASSISTAT software 7.7.

## 3. Results

### 3.1. Determination of Minimum Fungicide Concentration

The concentrations of the extract/fungus associations evaluated were determined using the Minimum Fungicide Concentration (MFC), and it was found to be safe to start at 20 mg/mL, which corresponds to half of the Minimum Inhibitory Concentration (MIC).

### 3.2. Immersion Test of R. microplus Females

Immersion tests on engorged females were conducted using plant extracts, either alone or in combination with the fungus *B. bassiana*. Results regarding the percentage reduction in oviposition, reduction in hatchability, reproductive efficiency, and product efficacy are presented in Table 1. Regarding the reduction in oviposition, treatments containing hexane and aqueous extracts yielded the best results. Among the treatments evaluated, SLh10 with fungus (+) resulted in a 26.32 ± 17.1% reduction in oviposition, while the SL water 10 with fungus (+) resulted in a reduction of 32.08 ± 16.3%. Regarding the reduction in hatchability, the greatest effect was observed with SL water 2.5 with fungus (+), which showed a reduction of 16.67 ± 15.0%.

**Figure 1:**
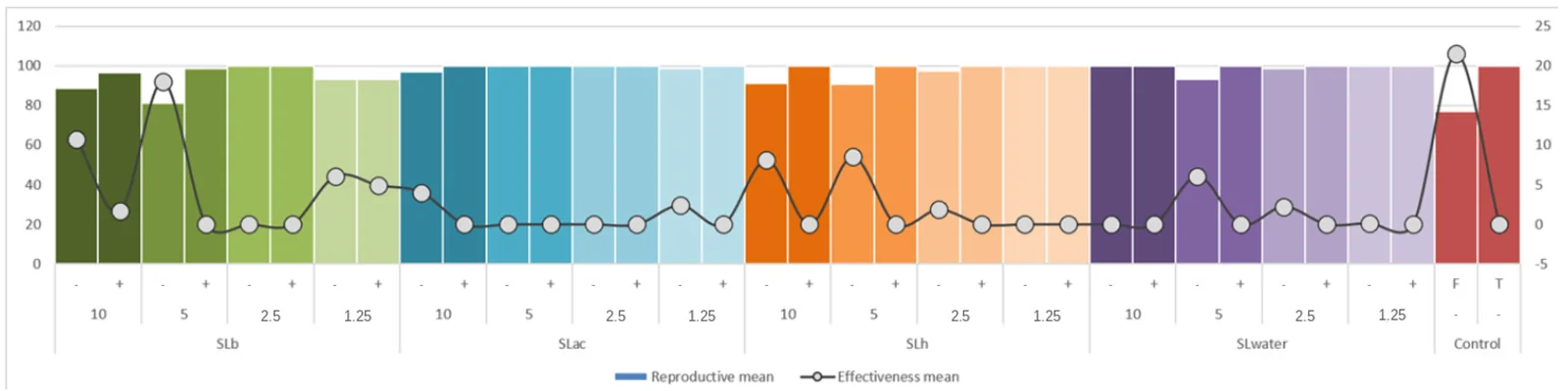
Effect of different *S. lentiscifolius* extract fractions and concentrations on reproductive and effectiveness parameters. SLb: *S. lentiscifolius* crude ethanolic extract; SLac: *S. lentiscifolius* ethyl acetate extract; SLh: *S. lentiscifolius* hexane partition; SLwater: *S. lentiscifolius* aqueous partition. (−) only extract, (+) extract + fungus, (F) fungus control, and (T) Tween control.

**Figure 2:**
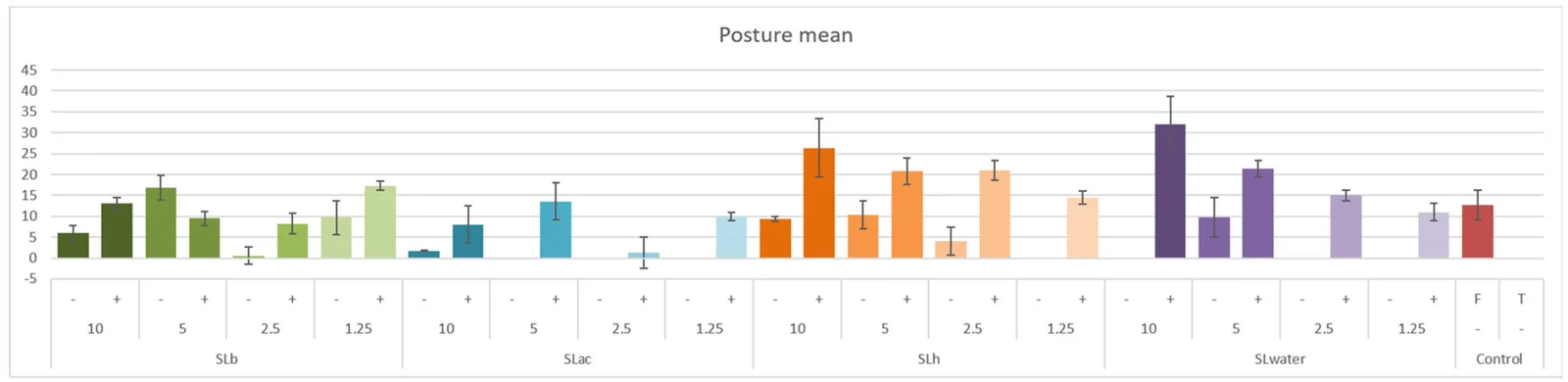
Effect of treatments with plant extracts, alone or combined with *B. bassiana*, on the reduction in oviposition by engorged females. SLb: *S. lentiscifolius* crude ethanolic extract; SLac: *S. lentiscifolius* ethyl acetate extract; SLh: *S. lentiscifolius* hexane partition; SLwater: *S. lentiscifolius* aqueous partition. (−) only extract, (+) extract + fungus, (F) fungus control, and (T) Tween control.

Figure 1 and Figure 2 present, in a simplified manner, the effect of different fractions of *S. lentiscifolius* associated (+) or not associated (−) with the fungus *B. bassiana*.

**Figure 3:**
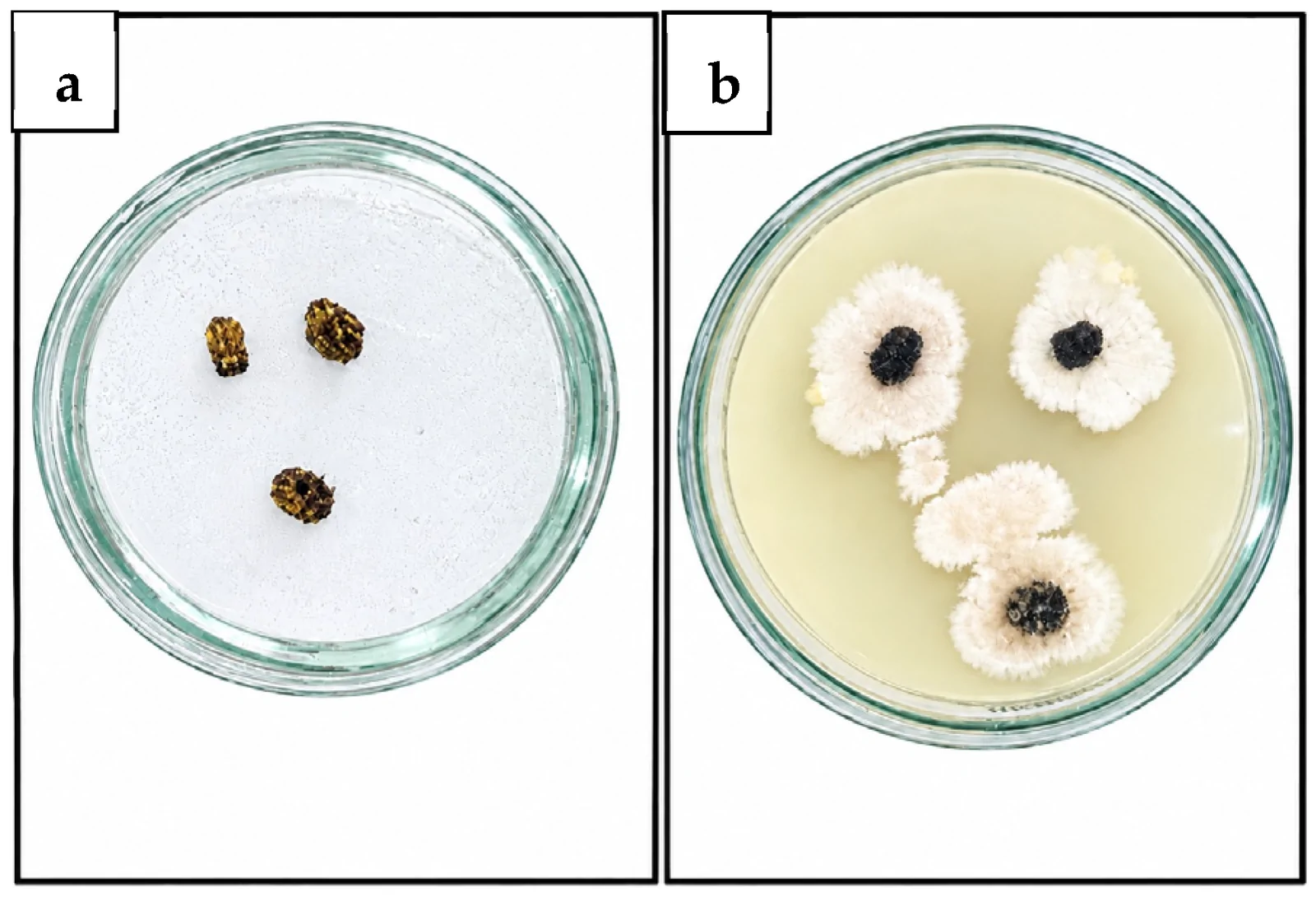
(**a**) Females of *R. microplus* SLb 10 mg/mL after oviposition in a plate with potato dextrose agar. (**b**) Females of *R. microplus* SLb 10 mg/mL with fungus after oviposition in a plate with potato dextrose agar.

After oviposition, the females exposed to the fungal suspension were transferred to culture plates containing a culture medium to assess fungal infection. All plates containing ticks previously immersed in the fungal suspension showed growth of *B. bassiana*, confirming fungal development on the treated ticks, as shown in Figure 3.

### 3.3. Sensitivity of Larval Packet Test on Impregnated Paper

The mortality of *R. microplus* larvae in the test on impregnated paper was significantly higher in treatments combined with *B. bassiana* (Table 2). The greatest effect was observed with SLb with fungus, showing 100 ± 0% mortality at 20 mg/mL and maintaining values above 90% at the other concentrations (98.63 ± 2.38%, 97.18 ± 2.60%, and 92.59 ± 4.94%). The SLac and SLh with fungus also increased mortality compared to the extracts alone, reaching 63.00 ± 6.76% and 50.81 ± 8.54%, respectively, at 20 mg/mL. Thus, SLb combined with fungus demonstrated the highest larvicidal efficacy among the treatments evaluated.

### 3.4. Larvae Immersion Test

In the results of larval immersion assay are presented in Table 3, the SLb with fungus treatment resulted in the highest mortality, reaching 100 ± 0% at 20 mg/mL, whereas SLb alone showed 75.73 ± 15.20%. For SLh, the combination with the fungus also increased mortality, reaching 68.25 ± 16.52% at 20 mg/mL, compared to 18.45 ± 9.30% for the extract alone. SLac with fungus treatment showed a maximum mortality of 57.19 ± 24.73% at the 20 mg/mL concentration. Overall, combining the extracts with the fungus *B. bassiana* increased larval mortality compared to the extracts alone, with SLb with fungus standing out as the most effective treatment.

## 4. Discussion

The growing demand for more sustainable livestock production has intensified the search for alternatives capable of reducing the environmental impacts associated with conventional farming systems. Among the main challenges are the reduction in greenhouse gas emissions, the optimization of water use, and the decrease in the use of synthetic drugs, which may persist in the environment and leave residues in products of animal origin. In this context, phytotherapy and biological control have emerged as promising strategies for veterinary applications. Although the use of plant-derived compounds in human and animal health has been extensively investigated, plant biodiversity still represents an important source of novel bioactive molecules. Likewise, entomopathogenic fungi have gained relevance as sustainable tools for parasite control. Based on these premises, the present study investigated the potential of *S. lentiscifolius* extractives, either alone or in association with *B. bassiana*, as an alternative for the control of cattle tick larvae, with the additional objective of reducing the concentrations of both plant extracts and fungal conidia required for biological activity.

The results obtained in the impregnated paper contact test [16] (FAO, 1971) demonstrated that the isolated extractives of *S. lentiscifolius* showed limited larvicidal activity, with the highest mortality being 10.53% for the crude ethanolic extract at 20 mg/mL. This low activity suggests that, under contact exposure conditions, the extractives alone may not be sufficiently effective to promote substantial larval mortality. However, when associated with *B. bassiana*, a marked increase in efficacy was observed, particularly for the combinations SLb + fungus, which achieved 100% mortality, and SLac + fungus, which achieved 63% mortality. Since the fungal control alone resulted in 41.65% mortality, these findings indicate that some extract–fungus combinations enhanced the larvicidal activity observed with *B. bassiana* alone.

A similar trend was observed in the larval immersion test [17], although this methodology allowed a clearer distinction among treatments. In the absence of the fungus, SLb and SLac were the most active extractives, reaching 75.73% and 37.96% mortality, respectively, at 20 mg/mL. These results indicate that the larvicidal potential of *S. lentiscifolius* depends not only on the type of extract or fraction evaluated, but also on the bioassay methodology employed. The superior performance observed in the immersion test may be related to the greater and more uniform exposure of larvae to the active compounds, in contrast to the impregnated paper test, where reduced contact time and compound volatilization may limit effectiveness. The association of extractives with *B. bassiana* in the immersion assay further reinforced the potential of this combined strategy. Although only the crude extract reached 100% mortality, the SLac and SLh fractions showed increased larvicidal activity when combined with *B. bassiana*, with mortality values 1.92 and 2.32 fold higher, respectively, than those observed for the fungal control alone at the highest concentrations tested. These findings are particularly relevant because studies addressing the combined use of plant extractives and entomopathogenic fungi for cattle tick control are still scarce. Therefore, the present results contribute to expanding current knowledge on integrated biological control strategies and suggest that combining fungal propagules with plant-derived compounds may improve efficacy even when lower concentrations of each agent are used.

This interpretation is consistent with the findings reported by Fantatto et al. (2022) [18] for *Achyrocline satureioides* associated with *B. bassiana* against *R. microplus*. In that study, the authors evaluated the activity of extracts and fractions in adult immersion, larval packet, and larval immersion assays, demonstrating that the association with the fungus enhanced the acaricidal effect of the plant-derived products. The hexanic partition of *A. satureioides* (ASh), at 5 mg/mL associated with the fungus, showed the highest efficacy against engorged females, reaching 43.3% and reducing larval hatchability. In addition, ASh associated with *B. bassiana* showed high larvicidal activity, with LC50 values of 6.23 mg/mL in the larval packet test and 2.65 mg/mL in the larval immersion test. Most importantly, the authors reported that the extract–fungus associations were able to reach 100% larval mortality, reinforcing the hypothesis that plant compounds can potentiate fungal action and improve tick control efficacy.

The enhanced activity observed at higher extractive concentrations may be partially explained by physicochemical aspects of the formulations. More concentrated solutions tend to present greater viscosity, which may favor adherence of the extractives to the larval cuticle and, consequently, facilitate the attachment and penetration of fungal conidia. In contrast, in less viscous solutions, spore adhesion may be reduced, compromising fungal infectivity. This hypothesis is consistent with the observation that some combinations performed better than the fungus alone, even when the plant extractives themselves showed limited isolated activity. Thus, the extractives may act not only through direct toxic effects but also by improving the conditions for fungal infection.

The performance of *B. bassiana* in the present study is also noteworthy when compared with previous reports. Barci et al. (2009) [19], using the *B. bassiana* strain IBCB66, reported 38.1% larval mortality at 10^7^ CFU/mL, whereas 100% mortality was achieved only at 10^9^ CFU/mL. In contrast, the fungal strain used in the present study produced 41.65% mortality at 10^6^ CFU/mL, indicating higher virulence at a lower concentration. This comparison suggests that strain 487 of *B. bassiana* may have greater pathogenic potential against cattle tick larvae than the strain evaluated by Barci et al. (2009) [19]. Furthermore, the improved results obtained when this strain was associated with *S. lentiscifolius* extractives reinforce the hypothesis that the interaction between fungal and plant-derived agents can significantly enhance larvicidal activity.

Another important aspect is the suitability of the larval immersion test for detecting the effects of combined treatments. Because this method minimizes the rapid volatilization of compounds and ensures direct contact between larvae and the test solutions, it may provide a more reliable assessment of the activity of plant extractives and their association with fungal conidia. This methodological aspect may explain why the immersion test produced more expressive results than the impregnated paper assay and highlights the importance of selecting appropriate bioassays when evaluating plant–fungus combinations.

Overall, the present findings demonstrate the promising potential of *S. lentiscifolius* extractives, particularly when combined with *B. bassiana*, for the sustainable control of *R. microplus* larvae. The increased mortality observed with the combined treatments indicates that the association of plant-derived compounds with the entomopathogenic fungus may enhance larvicidal activity and potentially allow the use of lower concentrations of the individual agents. Such an approach may offer economic and environmental advantages by reducing the amount of plant extract and fungal inoculum required for effective tick control. However, further studies are necessary to identify the bioactive compounds involved, elucidate the mechanisms responsible for the increased activity observed in the combined treatments, and determine the effectiveness of these associations under field conditions.

Regarding the safety of *S. lentiscifolius* extracts, Fantatto et al. (2025) [20] evaluated the ethanolic extract using *Galleria mellonella* as an alternative toxicity model and reported mortality below 20% after exposure to 2500 μg/mL for seven days. These findings provide preliminary evidence of low acute toxicity in the model evaluated. Nevertheless, results obtained in insect models cannot be directly extrapolated to cattle. Therefore, additional studies assessing cytotoxicity, dermal toxicity, and irritation, as well as controlled in vivo studies in mammals, are necessary to determine the safety of the extracts before their application to infested animals. In addition, determining the minimum effective concentration may help to minimize exposure of cattle to the extracts while maintaining their biological efficacy, thereby reducing the potential risk of adverse effects.

## 5. Conclusions

The present study demonstrated that *S. lentiscifolius* extractives, particularly when associated with *B. bassiana*, have promising potential for the control of *R. microplus* larvae. The combined treatments increased larval mortality compared with the extractives used alone, with the crude ethanolic extract associated with *B. bassiana* achieving the highest efficacy. These findings support the potential of plant–fungus combinations as an alternative strategy for tick control and suggest that effective activity may be achieved using lower concentrations of the individual agents. However, further studies are required to elucidate the mechanisms underlying the observed combined effects, identify the bioactive compounds involved, and evaluate the safety and efficacy of these associations under in vivo and field conditions.

## Figures and Tables

**Table 1:** Percentage of parameters evaluated in the immersion test of engorged females in different concentrations of different extracts of *S. lentiscifolius* associated and not associated with the fungus *B. bassiana* at a concentration of 10^6^ conidia/mL.

**SLb**	**Concentration** **mg/mL**	**% Oviposition** **Reduction**	**% Hatchability Reduction**	**% Reproductive** **Efficiency**	**% Product Effectiveness**
	10 (−) 10 with fungus (+)	5.98 ± 4.3 13.03 ± 3.7	5.84 ± 6.5 1.11 ± 6.1	88.65 ± 6.4 96.51 ± 11.6	10.69 ± 6.4 1.64 ± 11.8
	5 (−) 5 with fungus (+)	16.81 ± 7.1 9.52 ± 4.1	2.75 ± 3.1 7.04 ± 5.2	81.37 ± 9.3 98.54 ± 9.7	18.02 ± 9.3 0 ± 0
	2.5 (−) 2.5 with fungus (+)	0.57 ± 4.9 8.27 ± 6.1	0.34 ± 0.6 7.04 ± 0	100 ± 0 100 ± 0	0 ± 0 0 ± 0
	1.25 (−) 1.25 with fungus (+)	9.69 ± 10 17.29 ± 2.7	0 ± 0 12.22 ± 2.2	93.23 ± 3.8 93.27 ± 6.9	6.08 ± 8.6 4.93 ± 6.9
**SLac**	10 (−) 10 with fungus (+)	1.76 ± 3 8.02 ± 10.8	3.03 ± 1.1 3.70 ± 3.3	96.87 ± 2 100 ± 0	4.02 ± 9 0 ± 0
	5 (−) 5 with fungus (+)	0 ± 0 13.53 ± 10.9	1.01 ± 1.1 4.07 ± 3.5	100 ± 0 100 ± 0	0 ± 0 0 ± 0
	2.5 (−) 2.5 with fungus (+)	0 ± 0 1.25 ± 9.3	2.36 ± 1.5 0 ± 0	100 ± 0 100 ± 0	0 ± 0 0 ± 0
	1.25 (−) 1.25 with fungus (+)	0 ± 0 10.03 ± 2.4	2.69 ± 2.1 0 ± 0	98.49 ± 1 100 ± 0	2.41 ± 4.5 0 ± 0
**SLh**	10 (−) 10 with fungus (+)	9.40 ± 1.4 26.32 ± 17.1	0 ± 0 4.07 ± 14	91.21 ± 1.9 100 ± 0	8.11 ± 1.9 0 ± 0
	5 (−) 5 with fungus (+)	10.26 ± 8.2 20.80 ± 7.71	0 ± 0 0 ± 0	90.80 ± 5.9 100 ± 0	8.52 ± 6 0 ± 0
	2.5 (−) 2.5 with fungus (+)	3.99 ± 8.1 21.05 ± 5.8	0 ± 0 1.48 ± 2.7	97.41 ± 9 100 ± 0	1.87 ± 9.1 0 ± 0
	1.25 (−) 1.25 with fungus (+)	0 ± 0 14.54 ± 3.8	0 ± 0 0 ± 0	100 ± 0 100 ± 0	0 ± 0 0 ± 0
**SLwater**	10 (−) 10 with fungus (+)	0 ± 0 32.08 ± 16.3	1.35 ± 2.2 7.41 ± 13.8	100 ± 0 100 ± 0	0 ± 0 0 ± 0
	5 (−) 5 with fungus (+)	9.69 ± 11.6 21.30 ± 4.8	0 ± 0 9.63 ± 1.6	93.23 ± 11.6 100 ± 0	6.08 ± 4.1 0 ± 0
	2.5 (−) 2.5 with fungus (+)	0 ± 0 15.04 ± 3.2	3.37 ± 4.0 16.67 ± 15	98.67 ± 9.6 100 ± 0	2.23 ± 2.1 0 ± 0
	1.25 (−) 1.25 with fungus (+)	0 ± 0 11.03 ± 5	5.72 ± 4.5 1.48 ± 5.5	100 ± 0 100 ± 0	0.17 ± 8.01 0 ± 0
Fungus Control(F)		12.78 ± 8.7	10 ± 2.9	77.02 ± 2.2	21.50 ± 12
Tween^®^ Control(T)		0 ± 0	1 ± 2.2	100 ± 0	0 ± 0

SLb: *S. lentiscifolius* crude ethanolic extract; SLac: *S. lentiscifolius* ethyl acetate extract; SLh: *S. lentiscifolius* hexane partition; SLwater: *S. lentiscifolius* aqueous partition. (−) only extract, (+) extract + fungus, (F) fungus control, and (T) Tween control.

**Table 2:** Percentage mortality of *R. microplus* larvae in contact with papers impregnated with *S. lentiscifolius* extractives of different polarities, associated and not associated with the fungus *B. bassiana*.

Concentration mg/mL	SLb (Mortality %)	SLac (Mortality %)	SLh (Mortality %)
20	10.53 ^c^ ± 16.88	1.17 ^e^ ± 1.51	9.53 ^c^ ± 9.60
20 with fungus	100 ^a^ ± 0	63 ^b^ ± 6.76	50.81 ^b^ ± 8.54
10	0.72 ^c^ ± 0.70	0.38 ^e^ ± 0.65	5.81 ^c^ ± 6.95
10 with fungus	98.63 ^a^ ± 2.38	37.66 ^cd^ ± 7.33	49.96 ^b^ ± 5.44
5	0.18 ^c^ ± 0.30	0 ^e^ ± 0	2.58 ^c^ ± 1.75
5 with fungus	97.18 ^a^ ± 2.60	49.17 ^bc^ ± 6.66	40.76 ^b^ ± 7.48
2.5	0 ^c^ ± 0	0 ^e^ ± 0	1.55 ^c^ ± 1.94
2.5 with fungus	92.59 ^a^ ± 4.94	30.22 ^d^ ± 9.33	44.19 ^b^ ± 4.71
Negative Control	0 ^c^ ± 0	0 ^e^ ± 0	0 ^d^ ± 0
Positive Control	100 ^a^ ± 0	100 ^a^ ± 0	100 ^a^ ± 0
Fungus Control	41.65 ^b^ ± 3.7	41.65 ^c^ ± 3.7	41.65 ^c^ ± 3.7

SLb: *S. lentiscifolius* crude ethanolic extract; SLac: *S. lentiscifolius* ethyl acetate extract; SLh: *S. lentiscifolius* hexane partition. The Tukey test at a 5% probability level was applied, and the standard deviation was calculated. Means followed by the same letter do not differ statistically from each other. Positive control: Cypermethrin (15%), Chlorpyrifos (25%), Citronella (0.1%). Fungus control: *B. bassiana* only.

**Table 3:** Percentage mortality of larvae immersed in extractives of *S. lentiscifolius* of different polarities, associated or not with the fungus *B. bassiana*.

Concentration mg/mL	SLb (Mortality %)	SLac (Mortality %)	SLh (Mortality %)
20	75.73 ^b^ ± 15.20	37.96 ^cd^ ± 17.65	18.45 ^de^ ± 9.30
20 with fungus	100 ^a^ ± 0	57.19 ^bc^ ± 24.73	68.25 ^bc^ ± 16.52
10	7.36 ^e^ ± 1.84	5.08 ^e^ ± 2.07	2.13 ^f^ ± 2.54
10 with fungus	42.50 ^c^ ± 12.61	23.47 ^de^ ± 0.65	43.47 ^bcd^ ± 12.64
5	1.26 ^e^ ± 0.44	0.37 ^e^ ± 0.64	2.47 ^f^ ± 4.27
5 with fungus	26.31 ^cd^ ± 10.49	12.21 ^de^ ± 1.35	38.04 ^cde^ ± 4.76
2.5	3.43 ^e^ ± 4.46	1.75 ^e^ ± 3.03	0 ^f^ ± 0
2.5 with fungus	24.84 ^cd^ ± 6.53	1.86 ^de^ ± 4.19	28.09 ^de^ ± 15.54
Triton^®^ Control	5.24 ^d^ ± 0.31	5.24 ^d^ ± 0.31	5.24 ^d^ ± 0.31
Positive Control	100 ^a^ ± 0	100 ^a^ ± 0	100 ^a^ ± 0
Fungus Control	64.49 ^b^ ± 1.21	64.49 ^b^ ± 1.21	64.49 ^b^ ± 1.21

SLb: *S.* crude ethanolic extract; SLac: *S. lentiscifolius* ethyl acetate extract; SLh: *S. lentiscifolius* hexane partition. The Tukey test at a 5% probability level was applied, and the standard deviation was calculated. Means followed by the same letter do not differ statistically from each other. Positive control: Cypermethrin (15%), Chlorpyrifos (25%), Citronella (0.1%). Fungus control: *B. bassiana* only.

## Data Availability

The datasets generated and analyzed during the study “Combined effect of the association of extractives from *Schinus lentiscifolius* to the fungus *Beauveria bassiana* against the tick *Rhipicephalus microplus*” are available from the corresponding author, Rafaela Fantatto, upon request.

## Abbreviations

ANOVA	Analysis of Variance
BOD	Biochemical Oxygen Demand
CAPES	Coordination for the Improvement of Higher Education Personnel
CEUA	Committee on Ethics in the Use of Animals
CNPq	National Council for Scientific and Technological Development
CPPSE	Southeastern Livestock Research Center
CPPSUL	Southern Livestock Research Center
FAO	Food and Agriculture Organization
FAPESP	São Paulo Research Foundation
MFC	Minimum Fungicidal Concentration
MIC	Minimum Inhibitory Concentration
PDA	Potato Dextrose Agar
RPMI	Roswell Park Memorial Institute
SISGEN	National System for the Management of Genetic Heritage and Associated Traditional Knowledge
